# Potential Use of Quantum Dots in Flow Cytometry

**DOI:** 10.3390/ijms9122622

**Published:** 2008-12-17

**Authors:** Raquel Ibáñez-Peral, Peter L. Bergquist, Malcolm R. Walter, Moreland Gibbs, Ewa M. Goldys, Belinda Ferrari

**Affiliations:** 1 Department of Chemistry and Biomolecular Sciences, Macquarie University, Sydney, Australia. E-Mails: peter.bergquist@vc.mq.edu.au (P. B.); mgibbs@bio.mq.edu.au (M. G.); b.ferrari@unsw.edu.au (B. F.); 2 Department of Molecular Medicine & Pathology, University of Auckland Medical School, Auckland, New Zealand; 3 Department of Earth Sciences, Macquarie University, Sydney, Australia. E-Mail: malcolm.walter@unsw.edu.au; 4 Department of Physics, Macquarie University, Sydney, Australia. E-Mail: goldys@physics.mq.edu.au; 5 School of Biotechnology and Biomolecular Sciences, University of New South Wales, Sydney, Australia

**Keywords:** Quantum dots, flow cytometry, fluorescent detection, detection limit, autofluorescence

## Abstract

QDs may offer significant advantages in environmental and bead-based applications where the target cells need to be discriminated above background fluorescence. We have examined the possible applications of QDs for flow cytometric measurements (FCM) by studying their excitation - emission spectra and their binding to paramagnetic beads. We labelled beads with either QDs or a commonly-used fluorochrome (FITC) and studied their fluorescence intensity by FCM. Flow cytometric comparisons indicated that the minimum fluorophore concentration required for detection of QDs above autofluorescent background was 100-fold less than for FITC.

## 1. Introduction

Quantum dots (QDs) are inorganic crystalline nanoparticles made of semiconducting materials. QDs used for biological applications typically are composed of three different layers starting with a core, normally cadmium selenide (CdSe) that is coated with a semiconductor outer shell of zinc sulphide (ZnS). The core-shell ranges between 3 to 10 nm in size and the core size defines the fluorescence emission of the QDs. The third layer is composed of a polymer which solubilises the QDs while incorporating specific functional groups such as proteins and chemical compounds which allow specific binding to the desired target. The final size of the QDs is estimated to be up to 10–20 nm larger than their initial core [1].

Since Nie *et al*. and Alivisatos *et al.* published the first reports describing the use of QDs as fluorescent labels for biomolecules in 1998 [2, 3], interest in their applications has increased enormously. QDs are revolutionising many techniques in biological and biomedical analysis and their use has been reported in many applications including cell imaging [4, 5], multiplexed analysis of living cells [6, 7], imaging of entire subcellular structures [8], detection and targeting of specific cells [9], tracking cells over long periods of time [10] and labeling tissues and live microorganisms [11–14]. QDs are being used currently as a novel fluorophores because their physical and chemical properties [15–18] confer significant advantages over traditional dyes, such as brighter fluorescence [19, 20] and resistance to photobleaching [21]. Additionally, their unique optical properties include flexible excitation coupled with a narrow emission spectrum that enables simultaneous multiplexed detection and imaging using a single light source [15–17].

Multicolor optical coding using QDs potentially offers important advantages and applications for bead-based analyses in environmental microbiology that are not possible with conventional dyes. However, up to now, there have been only isolated reports of the applications of QDs for flow cytometry or for rapid readout of quantum dot-encoded mesoporous beads or biomolecules [22–24]. For example, the multiplexing capabilities of the QDs have been used to analyse the phenotype of multiple antigen-specific T-cell populations. The QDs were able to resolve up to 17 different fluorescence emissions [25]. QDs have been used for bacterial and pathogen detection in combination with flow cytometry [26, 27]. However, there are few reports of specific nucleotide probes being used for microbial identification using flow cytometry. Most reports have described QDs conjugated to specific antibodies for this purpose. The ability to use an oligonucleotide probe offers greater flexibility and cost benefits as compared to the production of antibodies specific to particular microorganisms.

We carried out studies to compare the fluorescence detection limit of QDs compared to the commonly-used dye, fluorescein isothiocyanate (FITC), to examine the applicability of QDs for flow cytometric detection. FITC is a small molecule (approx. 1.2 nm) that is excited over a narrow wavelength [18] but it is often used in biological applications such as the fluorescent-labelling of antibodies or other molecules to stain cells or cellular organelles [28]. We studied the binding of QDs to beads of a suitable size for detection since individual QDs are below the resolution of the flow cytometer. We established procedures to bind QDs and oligonucleotides modified with FITC to paramagnetic beads (Dynabeads). Characterization of the emission–excitation spectra of QDs showed that they can be excited by any wavelength shorter than that of their characteristic emission, but a decrease in fluorescence intensity was observed when excitation wavelengths go from 325 nm to 488 nm, the usual output of lasers used in most flow cytometers. Despite non-optimal excitation, we have demonstrated that when QDs are used as fluorophores to label beads, they still exhibit lower detection limits in flow cytometry in comparison to FITC.

The demonstration of the unique optical properties of the QDs has important implications for the study of environmental samples where microorganisms of interest need to be isolated away from the background debris which often causes the problem of high autofluorescence [29]. We investigated the effects of coupling an oligonucleotide labelled with FITC and QDs modified with biotin to paramagnetic beads (Dynabeads) for use as probes targeting specific DNA sequences from microorganisms. Dynabeads are of a larger size than bacteria and can be distinguished readily by FCM. Thus we were able to use them as a platform for detection. Our results revealed advantages of QDs over conventional dyes, even under non-optimal conditions, by excitation at shorter wavelengths as compared to UV wavelengths and opens up future opportunities for the expanded use of QDs in environmental applications using flow cytometry.

## 2. Experimental Section

### 2.1. Sources of Fluorescent Materials and Beads

Hops-Yellow CdSe/ZnS Evitags QDs (Evident Technologies Inc., Australia) were used in some preliminary experiments and were functionalized with amine groups. CdSe/ZnS Qdot™ QDs (Quantum Dot Corp., CA, USA) were functionalized either with biotin or streptavidin groups for binding to paramagnetic beads by the biotin-streptavidin interaction. Qdots™655 (red) were supplied in suspension with 50 mM borate buffer (pH 8.3). Fluorescent-dye conjugates to streptavidin were purchased from Invitrogen (Sydney, Australia). Dynabeads M-280 streptavidin are super-paramagnetic polystyrene beads of 2.8 μm in diameter (Dynal^®^ Biotech Pty. Ltd., Australia). They were supplied as a suspension containing 6.7 × 10^8^ Dynabeads per mL (10 mg/mL), dissolved in phosphate-buffered saline (PBS) pH 7.4. The binding capacity of the beads as claimed by the manufacturer was 1 mg of streptavidin-coated Dynabeads was able to bind 200 pmol of biotinylated oligonucleotide (single stranded).

### 2.2. Fluorescence Spectroscopy

Fluorescence measurements of Qdot^™^ 655-Biotin conjugates (QD655) were performed on a Perkin Elmer Luminescence Spectrometer LS 50B (Rowville, Victoria, Australia) equipped with a xenon discharge lamp. QD655 were diluted (1:1500) into dH_2_O in 10 mm Quartz cells (Hellma, NY, USA). The excitation spectrum was scanned between 310 nm and 490 nm. The emission spectrum was scanned between 500 nm and 700 nm at 1500 mm^−1^/sec scan speed.

### 2.3. Paramagnetic Beads

Dynabeads were used for all binding procedures at a stock concentration of 10 mg/mL and washed prior to use. Portions of bead solution (100 μL) were added to 1.5 mL Eppendorf tubes and placed in 1.5 mL Dynal MPC™ Magnet (Invitrogen, Mount Waverley, Australia) for 1–2 min to separate the beads from the original solvents and preservatives. The supernatant from each tube was removed carefully by aspiration with a pipette without removing the tube from the magnet. The tube was then removed from the magnet and 100 μL of BW buffer (10 mM Tris-HCl pH 7.5, 1 mM EDTA, 2.0 M NaCl) was added along the inside of the tube where the beads had been collected. The beads were resuspended in the same volume of BW buffer as the initial volume taken from the vial stock solution to keep the concentration of the beads constant. The washing step was repeated up to 3 times.

### 2.4. QD-biotin Labelling of Dynabeads

**Figure 1.**
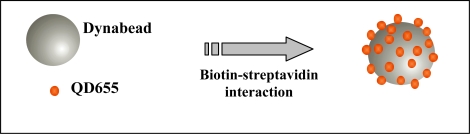
Schematic representation of Dynabeads labelled with QD655 by the biotin-streptavidin interaction.

Portions (5 μL) of the washed beads were transferred to Eppendorf tubes and resuspended in 200 μL of B Buffer (20 mM Tris pH 7.8, 1.0 M NaCl, 1 mM EDTA, 0.02% Triton X-100). Qdot^™^ 655 Biotin Conjugate (QD655) at a stock concentration of 2 μM were diluted (1:1000) in Qdot incubation buffer (2% BSA in 50 nM borate, pH 8.3 with 0.05% sodium azide). Binding was carried out by incubating the reactions at RT in the dark for 2 h with gentle rotation or occasional mixing. Figure 1 represents the binding of several QD655 to a single Dynabead. Unbound QDs were removed after incubation from the solution by washing the beads twice with 100 μL BW buffer, as described above. Finally, the QD-bead complexes were resuspended in 300 μL of BW buffer and stored in the dark at 4 °C for no more than 24 hours until flow cytometric analysis.

**Figure 2.**
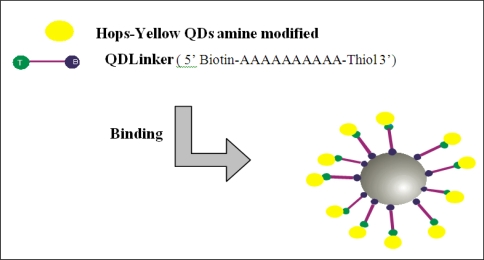
Schematic representation of QDLinker (a thiol-modified probe) coupled to amine-modified Hops-Yellow QDs by covalent interactions followed by the binding to Dynabeads by the biotin-streptavidin interaction.

A thiol-modified probe (QDLinker) was used for binding Hops-Yellow QDs to Dynabeads. QDLinker was a poly-A probe with a biotin modification at the 5’ end and thiol modification at the 3’ end (Sigma-Aldrich Pty Ltd. Australia). Figure 2 represents the binding of QDlinker to Hops-Yellow QDs by the covalent interaction between the thiol and the amine compounds, followed by binding to the Dynabeads by the biotin-streptavidin interaction. For activation, 450 μL of 1xPBS, 25 μL of QDLinker probe (0.1 mM) and 25 μL BMPA (N-β-Maleimidopropionic acid, 200 mM) were combined, followed by incubation for 2 h at RT. 3000 MWCO Microcon centrifugal filter devices (Millipore, USA) were used as indicated in the user’s manual to remove the excess of BMPA from the solution. A portion (70 μL) was transferred to a 1.5 mL Eppendorf tube containing 270 μL of dH_2_O, 50 μL of 10xPBS, 100 μL of amine modified Hops-Yellow QDs and 10 μL of EDC (1-Ethyl-3-(3-dimethyl-aminopropyl)carbodiimide hydrochloride, Sigma-Aldrich, USA) at a final concentration of 100 mg/mL. The solution was incubated at RT for up to 2 h and then 500 μL of 1 M Tris pH 7.4 was added to terminate the conjugation reaction. Excess unbound probe was removed using Microcon centrifugal devices (100, 000 MWCO). These filters discriminate between free probes and QDs. The final product was resuspended in 120 μL of 1xPBS and stored at 4 °C.

### 2.4. Binding FITC to Beads

**Figure 3.**
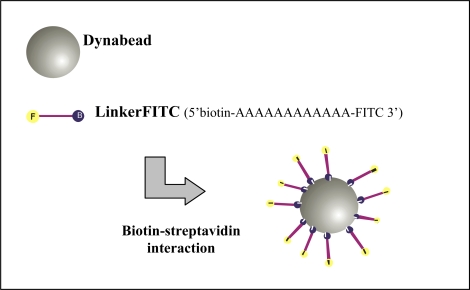
Schematic representation of LinkerFITC (biotinylated probe modified with FITC) bound to Dynabeads by the biotin-streptavidin interaction.

A poly-A oligonucleotide, LinkerFITC (5’ 7-AAAAAAAAAA-F 3’), with a biotin modification at the 5’ end and FITC modification at the 3’ end (Sigma-Aldrich Pty Ltd. Australia) was designed to enable the attachment of FITC to the Dynabeads and was used at a stock concentration of 100 μM. Figure 3 represents the binding of several LinkerFITC molecules to a single Dynabead. The beads were washed and prepared as described above. Portions (5 μL) of washed beads were transferred into Eppendorf tubes and resuspended in 200 μL of B Buffer. LinkerFITC was diluted (1:1000) in sterile MilliQ water and was used for preparation of serial dilutions. The binding procedures for LinkerFITC to beads were carried out as described for QDs. Following binding, the samples were washed twice with 100 μL of BW buffer and resuspended in 300 μL BW buffer. The LinkerFITC-bead complexes were stored in the dark for no more than 24 h at 4 °C until flow cytometric analysis.

### 2.5. Epi-fluorescence Microscopy

Washed, unlabelled beads and QD-bead complexes were imaged using an epi-fluorescence microscope (Olympus BH2-RFC, Olympus Corporation, N.Y. U.S.A) equipped with an HB100 mercury lamp for broad-band excitation. The filters selected for UV excitation were DM 400 and L-420 which reflect all light under 400 nm but allow the passage of wavelengths longer than 420 nm. The filter set selected for blue light excitation utilised BP 490, DM 500 and a supplementary exciter filter AFC 515 which reflects light from 490 nm to 500 nm onto the sample, collecting all the light above 515 nm. Samples were directly mounted onto slides by mixing 5 μL of sample with 5 μL of Citifluor AF-3 (Citifluor Ltd, UK) and sealing with nail polish to avoid desiccation and oxidation of the sample. Images were collected using a Nikon digital camera DXM1200F and Nikon ACT-1 version 2.62 software.

### 2.6. Flow Cytometric Analysis

**Figure 4.**
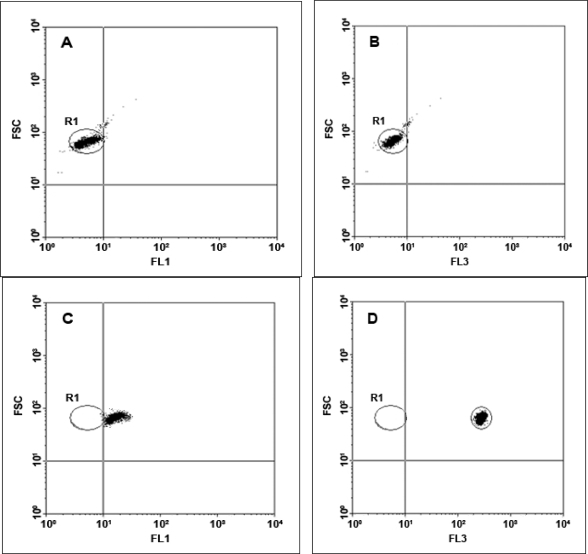

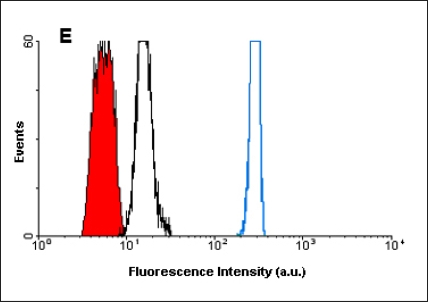
Flow cytometric analysis of bead-fluorophore complexes. A; Unlabelled beads on a dot-plot of FL1 (x-axis) versus FSC (y-axis) were positioned within the first decade of fluorescence. B: Unlabelled beads on a dot-plot of FL3 (x-axis) versus FCS (y-axis) were also positioned within the first decade. C: LinkerFITC-bead complexes on a dot-plot of FL1 (x-axis) versus FSC (Y-axis) at a concentration of 0.6 pmol of LinkerFITC. D: QDs-bead complexes observed on a dot-plot of FL3 (y-axis) versus FSC (y-axis) at a concentration of 0.6 pmol of QDs showed increased intensities over FITC. E: A histogram of the fluorescent intensity for QD-beads (blue line) and LinkerFITC-beads (black line) complexes versus unlabeled beads (red line) at the same fluorophore concentration in solution (0.6 pmol).

Flow cytometric analysis was performed using a BD FACSCalibur flow cytometer (BD Biosciences, Sydney, Australia), equipped with an argon ion laser (488 nm) for excitation. The detectors used were side scatter (SSC) with the voltage set at 150 V, forward scatter (FSC) E00, and three fluorescent detectors, with FL2 (yellow fluorescence emission) and FL3 (red fluorescence emission) set on 520 V and FL1 (green fluorescence emission) set on 474 V. A portion (10 μL) of washed, unlabelled beads diluted in 300 μL of W Buffer was prepared as a negative control that was used for instrument set-up for the fluorescence channels. The negative control was analyzed on a bivariate dot-plot of FSC versus SSC, thresholding on FSC. A region (R1) was constructed around the single population of the unlabelled beads. Bivariate dotplots of FSC versus FL1 (green) and FSC versus FL3 (red) were used for setting the FL1 and FL3 detectors by placing the negative control beads within the first fluorescence log decade until the median value from the negative control for both channels was equal (see Figure 4A and 4B). Green fluorescence from the LinkerFITC-bead complexes then was detected using the FL1 channel, with a 530/30 nm band-pass filter (see Figure 4C). Red fluorescence from the QD-bead complexes was recorded in the FL3 channel with a 650 nm long-pass filter (Figure 4D). A data file containing 3,000 events was recorded for each sample analyzed. The fluorescence intensity obtained from both LinkerFITC-bead complexes and QD-bead complexes was plotted on a histogram, showing that the fluorescent intensity of QD-bead complexes was higher than FITC at the same concentrations (Figure 4E).

### 2.7. Data Analysis

Data analysis was carried out with CellQuest software obtained from BD Biosciences (Sydney, Australia). For data analysis, FL1 and FL3 histograms were created by gating on the events falling within the defined region (R1). Samples of QD655-bead complexes were analysed on the FL3 histogram and the geo-mean value recorded (MFI value). The analysis program WinMDI version 2.8 was use for all data presentation of CellQuest data files and was obtained by downloading it from the World Wide Web (http://facs.scripps.edu/softaware.html).

### 2.8. Characterization of Probe Binding to QDs

Quantitative and qualitative methods were explored to investigate the number of molecular probes that could be attached to a single QD. Hops-Yellow QDs were used in this assay for probe binding. A quantitative method was developed based on the absorption spectra of both the QDs and the probes. The QDs and the molecular probes have different spectral signatures and hypothetically, two distinct peaks should be observed when studying the spectra of QDs with bound probes. One peak should correspond to the absorption of the QDs and a second peak should correspond to the absorption of the probes. Thus it should be possible to quantify the number of probes bound per QD from the spectral signatures. However, the broad absorption spectrum of the QDs resulted in high background noise especially in the 200 – 300 nm region, making it impossible to distinguish any distinctive peak in this region. Therefore, the absorption peak of the probes could not be detected (data not shown).

Gel electrophoresis was used as a qualitative method to determine the successful binding of the molecular probes to QDs. The size and the negative charge of the surface of the QDs made them suitable for gel electrophoretic analysis. Free QDs run faster through the gel as compared to QDs with bound molecular probes and they migrated more slowly than free oligonucleotide probes. Typical results from the gel electrophoretic analysis of QDs bound to oligonucleotide probes are shown below (see Figure 7).

## 3. Results

### 3.1. Excitation - Emission Spectra of QDs

**Figure 5.**
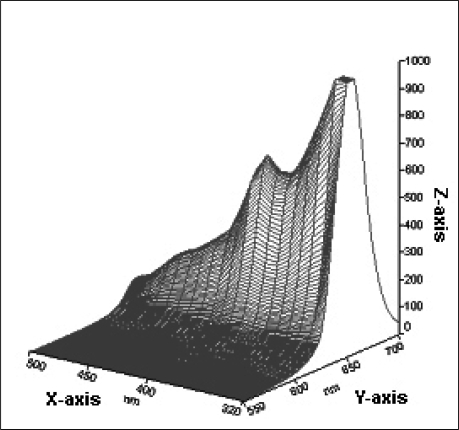
Excitation - emission spectra of red QDs. Z-axis: fluorescence intensity in arbitrary units. X-axis: Excitation wavelengths (nm). Y-axis: Emission wavelengths (nm). The emission peak was observed at 655 nm for all excitation wavelengths examined.

Qdot^™^ 655 Biotin conjugates were excited between 310 nm to 490 nm using a luminescence spectrometer. The emission spectrum was detected between 500 nm to 700 nm. The QDs analyzed exhibited maximum fluorescent emission at 655 nm (Figure 5). The results indicated that QDs remained fluorescent under all the excitation wavelengths examined. A 4-fold increase in fluorescence intensity was observed at short excitation wavelengths (UV = 320 nm) compared with the longer excitation wavelength (488 nm) that is commonly used in flow cytometers.

### 3.2. Fluorescence Emission of QDs

**Figure 6.**
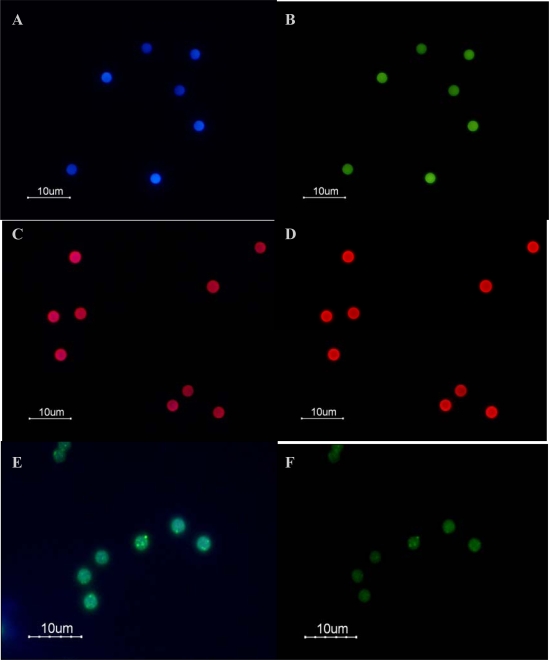
Images of unlabelled beads and QD-bead complexes observed under UV and blue light by epi-fluorescence microscopy. A: Unlabelled beads under UV light. B: Unlabelled beads under blue light. C: QD655-bead complexes under UV light exhibiting a bright red fluorescence. D: QD655-bead complexes under blue light also exhibit a shift to red fluorescence. E: Hops-Yellow QD-bead complexes under UV light exhibited green fluorescence and clusters of yellow fluorescence due to agglomeration of the QDs. F: Hops-Yellow QD-bead complexes under blue light also exhibit green and yellow fluorescence.

Samples were analyzed using several excitation–emission filters to capture UV and blue light for confirmation of successful binding by fluorescence. Analysis of the negative control revealed that the unlabelled beads emitted a low level of background autofluorescence (Figure 6A and 6B). Despite this observation, a significant increase in red fluorescence was observed after binding red QDs (QD655) to the Dynabeads under both filters examined, demonstrating the success of the binding procedure and the broad excitation spectrum of the QDs (Figure 6C and 6D). No clustering of the fluorescence on the surface of the beads was observed, indicating that the QD655 had not become agglomerated on areas of the bead surface. Consequently, we infer that the beads were bound successfully with a monolayer of QD655. A shift in fluorescence emission of the Dynabeads from blue to green after binding of Hops-Yellow QDs was observed under UV excitation (Figure 6E). However, Hops-Yellow QDs were found to form clusters on the surface of the Dynabeads, which resulted in agglomeration.

### 3.3. Qualitative Demonstration of the Binding of QDs to Dynabeads

**Figure 7.**
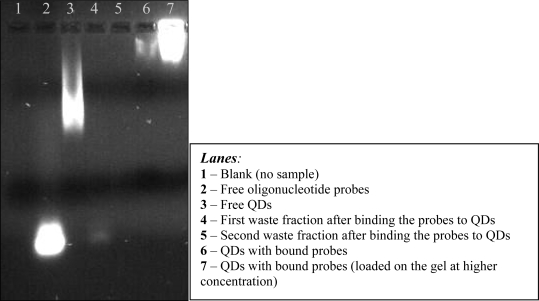
Free QDs, free oligonucleotide probes and bound probes bound to QDs run on a 1% agarose gel. Slower migration of the QDs with bound probes confirmed successful binding of the oligonucleotides.

Typical results from gel electrophoresis analysis of QDs with bound oligonucleotide probes are shown below (Figure 7). The size of free QDs allowed them to run successfully on an agarose gel (Figure 7, lane 3). QDs with bound probes were considerably larger and consequently, ran more slowly on the gel (Figure 7, lanes 6 and 7). The waste fractions were also analysed to ensure that both QDs and probes had bound during the procedure. The first waste fraction (Figure 7, lane 4) showed the presence of a small amount of unbound probe but the second waste fraction did not contain unbound probe or QDs (Figure 7, lane 5).

### 3.4. Flow Cytometric Measurements

The fluorescent intensity of Dynabeads labeled with different amounts of QD655 or LinkerFITC was analyzed by flow cytometry. In both cases, the flow cytometer was set up to ensure that the negative control exhibited the same mean value in both channels FL1 and FL3 (Figure 4A and 4B).

**Figure 8.**
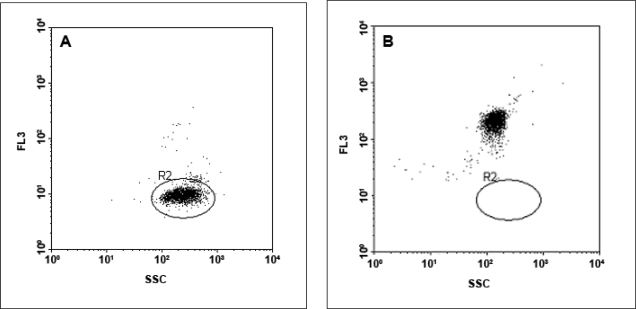
Flow cytometric analysis of QD655 bound to Dynabeads. Bivariate dot-plots defining log FL3 channel (y-axis) versus log SSC channel (x-axis) A; Unlabelled Dynabeads. A circular region (R2) was defined around the unlabelled Dynabeads. B; QD655 bound to Dynabeads. A significant increase in fluorescence emission by the complex confirmed successful binding of QD655 to the Dynabeads.

Dynabeads prior to and after binding to QD655 were analysed with a BD-FACSCalibur flow cytometer and the mean fluorescence intensity (MFI) was measured. Dynabeads bound to QD655 exhibited a substantial increase in the intensity of the fluorescent signal in FL3 (MFI: 199) compared to unlabeled Dynabeads (MFI: 10). This increase in fluorescence indicated positive binding between the QDs to the Dynabeads (Figure 8). The background fluorescence of the Dynabeads did not seem to affect the fluorescence emission of the QD655-Dynabead complexes. Hops-Yellow QDs-Dynabead complexes were also examined but did not show a fluorescent signal above the background fluorescence of the Dynabeads (data not shown).

**Figure 9.**
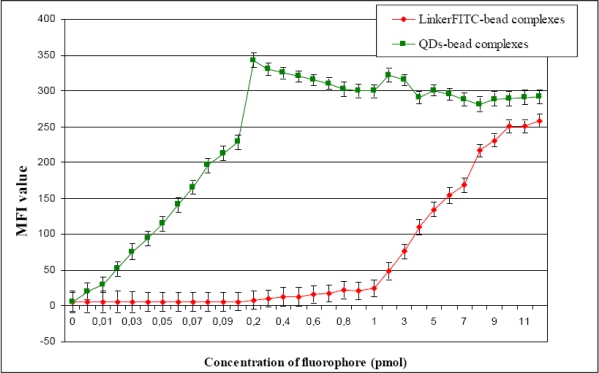
Fluorescent intensity of QD655-bead complexes versus the fluorescent intensity of LinkerFITC-bead complexes measured as MFI values by flow cytometry. X-axis: Concentration of QD655 and LinkerFITC in pmol bound to Dynabeads (5 μL beads, 10 mg/mL, per reaction). Y-axis: Median MFI value of each sample.

The fluorescent signals of Dynabeads labelled with both fluorophores (FITC and QD655) were analyzed by flow cytometry and the MFI values collected (Figure 9). The MFI value of each sample was obtained by defining an elliptical region around the centre of the main fluorescing population of Dynabeads (R2). Once the region was defined, the MFI value was obtained using the CellQuest software. The maximum binding capacity of beads for oligonucleotides probes, as indicated by the product data sheet, was 200 pmol of biotinylated oligonucleotides per one milligram of beads (Dynal Biotech) or 10 pmol of biotinylated probe per 5 μL of Dynabeads stock solution. However, the binding capacity of Dynabeads appeared to be different for the two fluorophores examined.

Dynabeads labelled with QD655 were observed to reach maximum binding capacity at significantly lower concentrations than FITC (Figure 9). The fluorescent signal of QD655 bound to Dynabeads increased exponentially until they reached their saturation point (0.2 pmol). However, LinkerFITC bound to the beads confirmed the stated bead commercial binding capacity as the maximum fluorescent intensity was observed at 10 pmol of LinkerFITC per 5 μL of beads (Figure 9). The MFI value of each reaction showed that QD655 bound to Dynabeads could be clearly discriminated above the negative control at amounts QDs as low as 0.01 pmol. By comparison, the minimum amount required to detect clearly LinkerFITC bound to Dynabeads above the negative control was at a much higher concentration of 1 pmol. Thus, the QD655-bead complexes exhibited a 100-fold increase in their minimum detection limit compared with FITC-bead complexes when using a 488 nm excitation source.

## 4. Discussion

Commercially-available QDs have been described as highly fluorescent particles with very broad excitation spectra and narrow and symmetric emission spectra, enabling multiple colours to be excitable by a single light source. Their behaviour has not been fully characterized although they have been used in a large number of biological applications. Moreover, commercial QDs are not spectroscopically standardized and their properties can be influenced by details of their synthetic history [30, 31]. Therefore, the physical and optical properties of commercial QDs were investigated by measuring their fluorescence emission over a wide range of excitation wavelengths.

The excitation and emission spectra properties of QD655 in solution were examined. Their fluorescent intensity decreased at long excitation wavelengths by an approximately 4-fold difference from 320 nm (UV light) to 490 nm (blue light). These results were in accordance with the broad absorption spectra reported previously [16]. The optical properties of QD655 in solution were found to be different to QD655 bound to Dynabeads. The fluorescent intensity of QD655 bound to the Dynabeads by the biotin-streptavidin interaction was found to be two-fold greater under UV light than under the blue light. Therefore, it was concluded that approximately 50% of the fluorescent emission was lost when the QDs were bound to the Dynabeads compared to free QDs (Ibáñez-Peral, unpublished thesis).

The fluorescent signal of Hops-Yellow QDs and FITC could not be determined by flow cytometry. Although both QDs examined were detectable in the same channel, the fluorescent signal of the Hops-Yellow QDs was lost after binding to the Dynabeads. Hops-Yellow QDs were functionalised with amine groups. It has been reported that solvent polarity and pH can effect the absorption spectra of organic compounds such as amine groups [32]. In addition, the molecular probes used were modified with thiol groups to allow covalent attachment to QDs. Thiol groups may be photocatalytically-oxidised making the QDs photochemically unstable and, consequently causing them to loose their absorption and emission efficiency [33]. Furthermore, the clusters of Hops-Yellow QDs observed under epi-fluorescence microscopy (Figure 6E) may be due to photooxidation. Photooxidation of the thiol groups has been reported to cause the formation of micelle-like structures around QDs, causing agglomeration. Therefore, the optical properties of Hops-Yellow QDs were influenced after binding to the Dynabeads. Further development of QDs synthesis is needed in order to obtain high-quality photostable QDs.

A single QD has approximately 7 active groups for the streptavidin-biotin binding reaction on its surface, as indicated by the manufacturer. Quantitative and qualitative methods were attempted to confirm this information by calculating the number of molecular probes that could be attached to a single QD. Spectrophotometric analyses were attempted to study the absorption spectra of QDs bound to molecular probes but their broad absorption spectra resulted in high backgrounds at low wavelengths, making it impossible to detect any distinctive peak attributable to the oligonucleotides. Although gel electrophoresis could not give an accurate number of probes bound to the QDs, these analyses were useful in verifying the binding procedures (Figure 7).

The binding capacity of the Dynabeads was found to be different for QDs and organic dyes. The maximum binding capacity of LinkerFITC to Dynabeads was reached in accordance with the claims made by the manufacturer. The maximum fluorescence intensity of QD655-bead complexes was observed at a much lower concentrations than for FITC. This reduction may be explained in terms of steric hindrance of fluorophore binding. The final size of commercial QDs is approximately 20 nm, while FITC is approximately 1.2 nm. This increase in size could severely affect probe binding. Potentially, this phenomenon could have significant consequences for many biological and biomedical applications where the samples studied have low binding capacities for their targets, and consequently, may make them undetectable by flow cytometry. The capability of QDs for specific target detection, coupled with lower detection and greater multiplexing capability using single light sources has been suggested to offer significant advantages over conventional organic dyes [34]. However, the results found here showed that the binding capacity of the QDs is considerably lower than organic dyes and thus may substantially reduce their capacity for target detection by flow cytometry.

The fluorescent signal of QD655 appeared to be higher than FITC but the comparison could not be quantified. QD655 was detected in the FL3 channel of the flow cytometer while FITC was detected in the FL1 channel. For comparative purposes, both fluorophores should have been analyzed with the same channel and experimental settings. Most methods used to quantify the fluorescence from unknown samples are based on converting the MFI values obtained by flow cytometry into Molecules of Equivalent Soluble Fluorochrome (MESF) units [35]. The MESF unit corresponds to the fluorescent intensity of a given number of pure fluorochrome molecules in solution. Even if both fluorophores could have been detectable in the same channel of the flow cytometer and had the same emission wavelength, such as Hops-Yellow QDs and FITC, their excitation wavelength would have had be different as well as their quantum efficiency. Therefore, a direct fluorescence comparison of FITC and Hops-Yellow QDs or different green QDs (e.g. QD525), using MESF units would not have been possible.

## 5. Conclusions

The unexpected results found when studying the physical and optical characteristics of QDs could potentially lead to difficulties when using them in biological applications. Many fundamental characteristics of their surface chemistry and physicochemical properties in varying situations appear to need standardization. Some important technical problems remain, particularly in defining and characterizing the surface coating chemistry. Ideally, QDs should maintain strong fluorescence without bleaching, quenching, or blinking. Further analyses aimed at studying the binding characteristics of QDs are required before they can be employed in experimental situations that require complex manipulations. In a further communication, we will describe the use of organic fluorophores and QDs in bead-based assays involving the PCR of genomic DNA for the identification of microorganisms in environmental samples (Ibáñez-Peral *et al*, in preparation).
